# Case report: Surgical treatment of an astrocytoma in the thoracic spinal cord of a cat

**DOI:** 10.3389/fvets.2023.1264916

**Published:** 2023-10-24

**Authors:** Koen M. Santifort, Shinji Tamura, Daniel R. Rissi, Guy C. M. Grinwis

**Affiliations:** ^1^IVC Evidensia Small Animal Referral Hospital Arnhem, Neurology, Arnhem, Netherlands; ^2^IVC Evidensia Small Animal Referral Hospital Hart van Brabant, Neurology, Waalwijk, Netherlands; ^3^Tamura Animal Clinic, Hiroshima, Japan; ^4^Athens Veterinary Diagnostic Laboratory, Department of Pathology, College of Veterinary Medicine, University of Georgia, Athens, GA, United States; ^5^Veterinary Pathology Diagnostic Centre, Faculty of Veterinary Medicine, Department of Biomedical Health Sciences, Utrecht University, Utrecht, Netherlands

**Keywords:** glioma, astrocytoma, feline, neoplasia, dorsal laminectomy

## Abstract

A 15-year-old spayed female domestic shorthaired cat was evaluated for chronic progressive paraparesis and proprioceptive ataxia. Neurological examination was consistent with a T3–L3 myelopathy. Plain thoracolumbar vertebral column radiographs and CT without intravenous contrast or myelography performed at another facility did not highlight any abnormalities. MRI of the thoracolumbar spinal cord identified an intraparenchymal space-occupying lesion extending from T10–T12. Surgery was performed to remove as much of the mass as possible, and to submit samples for histopathology. A dorsal laminectomy was performed over T9–T13. A midline myelotomy provided access to the mass, which was debrided with an intraoperative estimate of 80% removal. Histopathologic examination was consistent with a diagnosis of an astrocytoma. Post-operative treatment consisted of amoxicillin clavulanic acid, prednisolone, gabapentin, and additional analgesic medications in the direct post-operative period. Over the following 4 months, slow recovery of motor function was seen with continued physiotherapy. During the following 2 months, renal and cardiopulmonary disease were diagnosed and treated by other veterinarians. The cat was also reported to have lost voluntary movement in the pelvic limbs during this period, suggesting regression to paraplegia. Finally, 6 months post-surgery, the owner elected humane euthanasia. This is the second documentation of surgical treatment and outcome of an astrocytoma in the spinal cord of a cat.

## Introduction

The frequency of different types of feline spinal cord neoplasms varies according to the affected spinal cord compartment (1–5). While lymphomas are one of the most commonly diagnosed extradural tumors and is the most frequent type of neoplasia affecting the spinal cord of cats (1, 2), intradural/extraparenchymal neoplasms (such as meningiomas and nerve sheath tumors) and intraparenchymal neoplasms (including gliomas) are infrequently reported (4, 5). Spinal cord gliomas consist mainly of astrocytomas and oligodendrogliomas (5).

To the authors’ knowledge, there is only one case report of surgical treatment of feline spinal cord glioma (6, 7). As gliomas in the feline spinal cord are rare, it is unlikely that large case series discussing the outcomes of surgical treatment thereof will become available the near future. Therefore, single case reports may provide some information to help guide clinical decision-making regarding the surgical treatment of feline spinal cord gliomas. In this case report, we describe the clinical findings, diagnostic imaging features, surgical treatment, and outcome in a cat with an astrocytoma in the thoracic spinal cord.

## Case description

A 15-year-old spayed female domestic shorthaired cat was evaluated for chronic progressive paraparesis and proprioceptive ataxia. Previous history included repeat tail-amputation surgery, initially indicated due to a traumatic tail wound, with wound infections 5 months earlier. At the time of referral, these issues had resolved. The paraparesis and ataxia were more recent in onset, having been noticed about a month earlier. Treatment with robenacoxib (1.5 mg/kg q24h per os) had been prescribed due to suspected thoracolumbar hyperesthesia, with some positive effect. However, neurological deterioration was noticed. Gabapentin (10 mg/kg q8h per os) was prescribed but discontinued due to sedation side-effects. Plain thoracolumbar vertebral column radiographs and computed tomography (CT) without intravenous contrast or myelography performed at another facility did not highlight any abnormalities.

Neurological examination revealed spastic ambulatory paraparesis, left worse than right, with proprioceptive ataxia. Crossed extensor reflexes and patellar hyperreflexia were evident in both pelvis limbs. The cutaneous trunci reflex was absent caudally from the thoracolumbar junction. Mild thoracolumbar hyperesthesia (caudal thoracic region) was noticed on palpation. These findings were deemed consistent with a T3-L3 myelopathy.

Hematology and blood biochemistry were unremarkable. The cat was anesthetized and positioned in dorsal recumbency for a magnetic resonance imaging (MRI) of the thoracolumbar spinal cord (1.5 T Canon Vantage Elan). The following sequences were performed: T2W sagittal, T1W sagittal, STIR sagittal, STIR dorsal, T2W transverse, T1W transverse, T2* GRE transverse, T1W sagittal post-contrast, T1W transverse post-contrast, and 3D T1W magnetization prepared - rapid gradient echo (MPRAGE).

**Figure 1:**
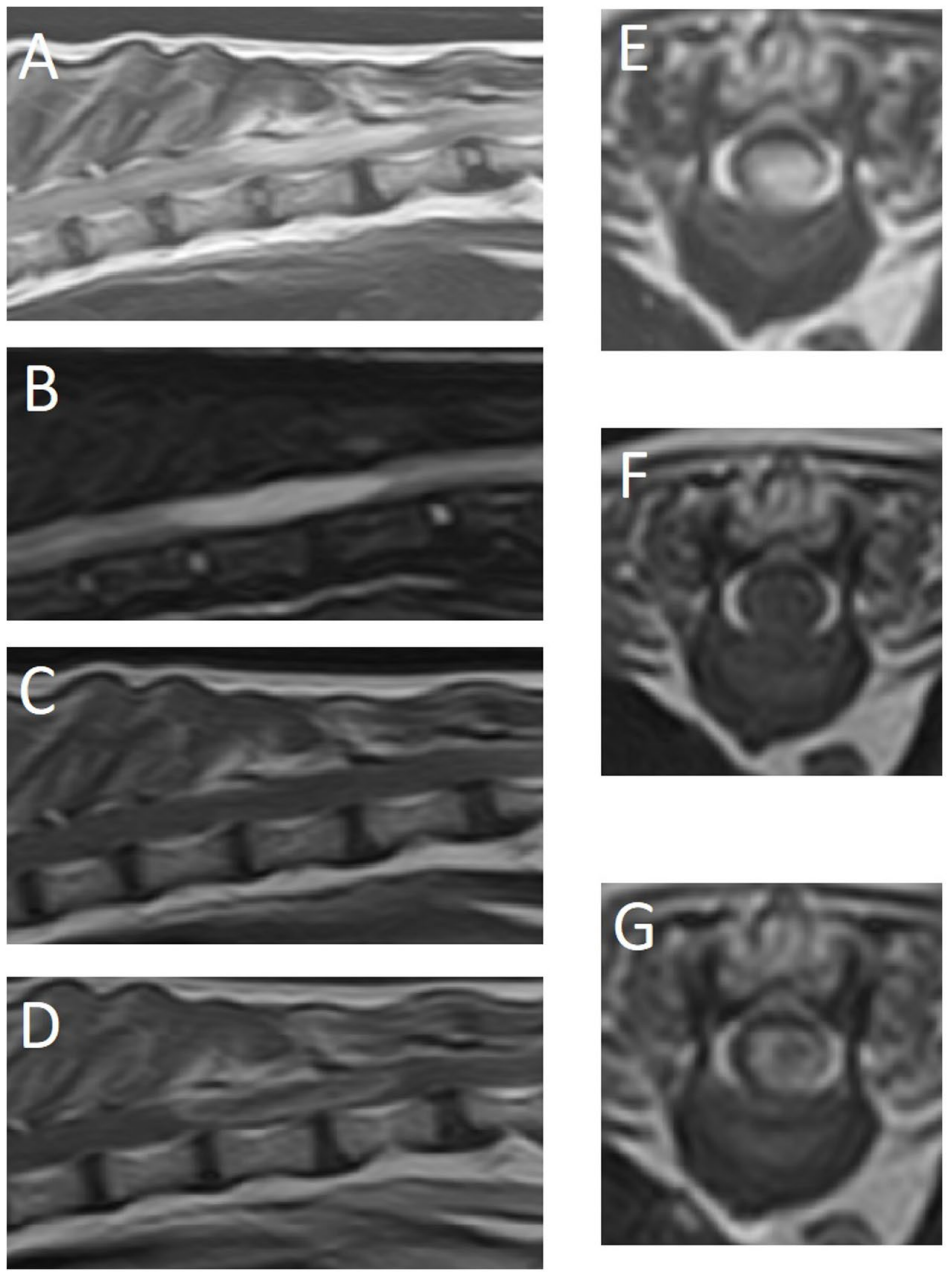
Magnetic resonance images of the thoracolumbar spinal cord and surrounding structures. **(A)** T2-weighted sagittal plane, **(B)** short-tau inversion recovery (STIR) sagittal plane, **(C)** T1-weighted sagittal plane, **(D)** T1-weighted post-contrast sagittal plane, **(E)** T2-weighted transverse plane at the level of T11, **(F)** T1-weighted transverse plane at the level of T11, **(G)** T1-weighted post-contrast transverse plane at the level of T11.

MRI identified an intraparenchymal space-occupying mass extending from T10–T12 (Figure 1). The lesion was somewhat left-sided, well-defined, and elliptical (2.4 cm length x 0.4 cm height x 0.4 cm width). It was homogeneously hyperintense on T2-weighted (T2W) and short-tau inversion recovery (STIR) images, and isointense on T1W images. Moderate, thick ring enhancement was noted, with minimal to no enhancement of the lesion center. There was no evidence of meningeal enhancement nor other extradural or intradural lesions. Cranially and caudally to the mass, there was increased intraparenchymal T2W signal intensity, reaching the level of T1 cranially and L5 caudally. On the cranial aspect, there was also mild dilation of the central canal. No vertebral lesions were seen. The intervertebral discs were hypointense in T2W (mild to moderate degree), indicating some level of intervertebral disc degeneration. Based on these findings, the main differential diagnoses included inflammation (e.g., intraparenchymal abscess) or neoplasia (e.g., glioma, lymphoma, or ependymoma).

A cerebrospinal fluid (CSF) tap yielded a total nucleated cell count of 24 cells/μL (reference range < 5) and semiquantitative increased protein levels (on a urine dipstick, 100 mg/dL). Cytological examination was non-diagnostic due to poor cell preservation (impression of a mixed pleocytosis). A fine-needle aspirate was attempted at the site of the mass lesion (T11–12), but results were non-specific (granular eosinophilic material, red blood cells, neutrophils, and lymphocytes). Given the history of wound healing complications after tail-amputation with bacterial infections, treatment was initiated with amoxicillin clavulanic acid (15 mg/kg q12h per os) and prednisolone (0.7 mg/kg q24h per os). Meanwhile, CT images were reviewed and abdominal ultrasound was performed, but no evidence of neoplasia was found elsewhere. The patient had shown clear signs of deterioration over the following week. Treatment options were discussed and surgery was elected with the aim to remove as much of the mass as possible and to submit tissue samples for histopathology. Surgery was scheduled for 2 weeks after initial presentation. By that time, complete spastic paraplegia with intact nociception was evident. Urinary incontinence (upper motor neuron bladder) was present.

**Figure 2:**
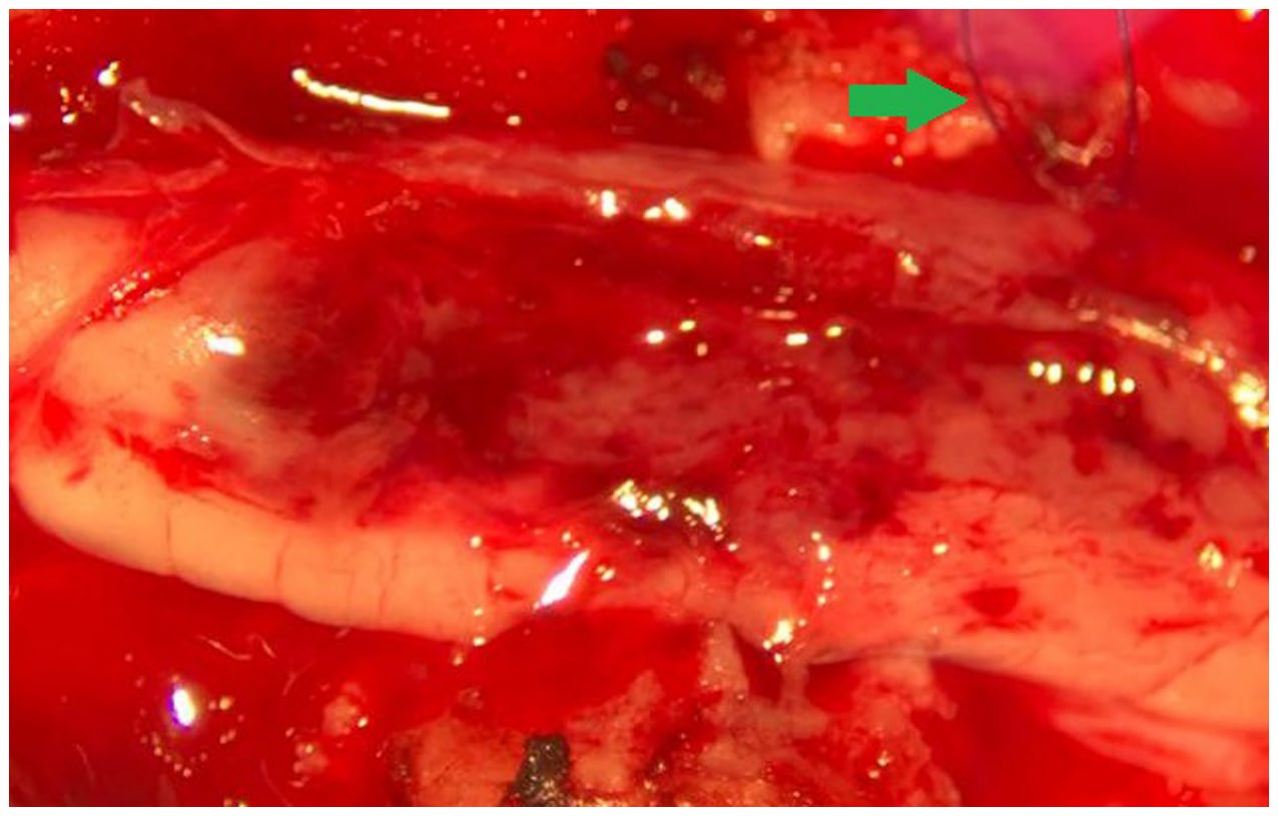
Intraoperative photograph (via operating microscope) showing the macroscopic characteristics (color, margination) of the thoracic spinal cord astrocytoma. The green arrow points to the stay suture in the dura mater on the right side of the spinal cord. Cranial is left in the image.

The cat was anesthetized and positioned and stabilized in sternal recumbency. Perioperative cefazoline (20 mg/kg, q6h IV) was administered for antibiosis. A midline incision was performed over the T9-L1 vertebrae. After removal of the T10, T11, and T12 spinous processes, a dorsal laminectomy was performed from the caudal lamina of T9 and cranial T13 with a burr and Kerrison rongeurs. The spinal cord contained within the dura mater at the location of T12 was darkened dorsolaterally on the left. Using a surgical microscope, a dorsal midline incision into the dura mater was performed. Stay sutures were positioned in the meninges, which were reflected to the sides. Specifically, the dura mater was incised in an H pattern, also aimed at relieving intradural pressure. The dorsal median sulcus was evident cranially and caudally to the lesion and a midline myelotomy was made along its course. Malacic looking tissue from the center of the mass was removed with careful suction, and the intraparenchymal mass was removed in small fragments that detached from the surrounding parenchyma. A poor plane of cleavage of presumably affected tissue from healthy tissue was noticed, suggestive of an infiltrative nature. Some tension was put on the dura mater stay sutures to facilitate careful exploration and removal of the intramedullary mass with microsurgical equipment, and the spinal cord was lifted slightly dorsally in the process. The dark red mass looked to be invasive into, and blending into normal tissue at the borders (Figure 2). Approximately 80% of the mass could be removed in a piecemeal fashion, from the inside-out based on color and consistency. This was done with care, making use of gelatin sponge spears and microsurgical instruments. Complete excision could not be performed, largely due to difficulty in distinguishing the margins between neoplastic tissue and edematous spinal cord parenchyma. There were no hemorrhages at the time of closure. The dura mater was not sutured. Routine closure of the epaxial musculature, fascia, subcutis, and dermis was performed.

**Figure 3:**
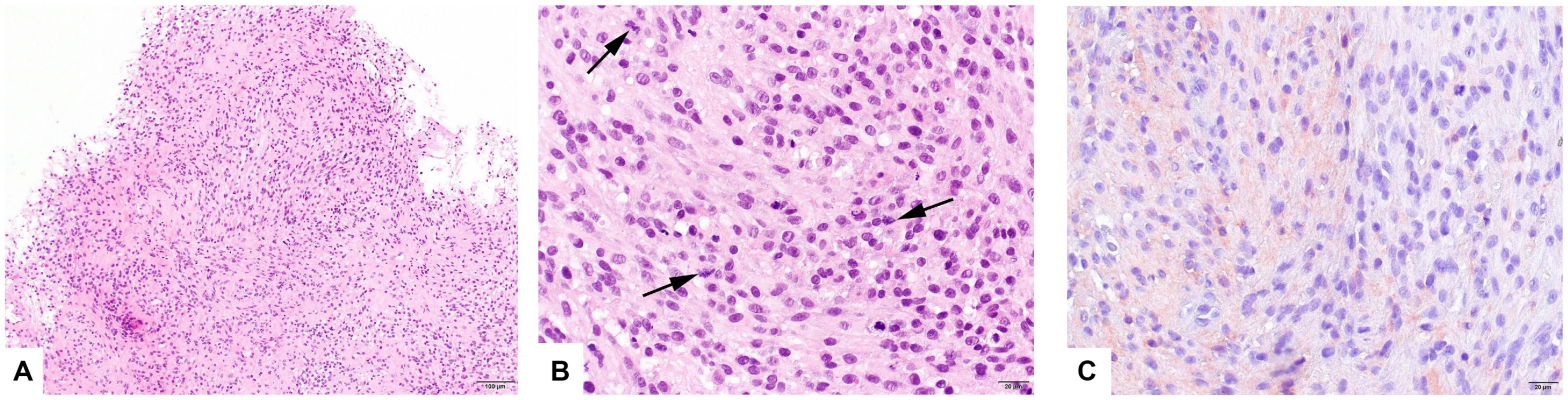
Histology of surgically acquired samples of the intraparenchymal mass lesion. Sheets of neoplastic cells, partly arranged in haphazardly oriented bundles **(A)**. The neoplastic cells show moderate anisokaryosis. Mitotic figures (arrows) are frequently seen **(B)**. Glial fibrillary acidic protein (GFAP) immunohistochemistry (IHC) highlighted mild–moderate cytoplasmic positivity throughout the tissue samples **(C)**.

Histological examination of hematoxylin and eosin stained formalin-fixed paraffin-embedded tissue section revealed a pleomorphic population of neoplastic cells with eosinophilic cytoplasm and indistinct cell borders (Figure 3). The most part of the neoplastic cell population was elongated and arranged in somewhat haphazardly arranged bundles (Figure 3A). The nuclei were oval-to-round, variable in size, and primarily eccentric, with 1–3 nucleoli and finely stippled chromatin. Throughout the tissue samples, mitotic figures were seen, with a highest density of 23 mitotic figures in 2.37 mm^2^ (Figure 3B). Glial fibrillary acidic protein (GFAP) immunohistochemistry (IHC) highlighted mild–moderate cytoplasmic positivity throughout the tissue samples. These findings were consistent with a diagnosis of an astrocytoma in the thoracic spinal cord. Based on these findings, it was concluded that previous tail amputation surgeries and wound healing complications were unrelated to the spinal cord pathology.

Post-operative treatment consisted of amoxicillin clavulanic acid (15 mg/kg q12h per os, discontinued a week later when histological report was available), gabapentin (10 mg/kg q8h), and prednisolone (0.7 mg/kg q24h per os). Methadone boluses were given as required based on pain scores (0.1–0.3 mg/kg). Ketamine continuous rate infusion was continued after surgery (employed as part of the anesthetic protocol) and discontinued 36 h later. The cat was discharged 4 days post-surgery.

Nociception was present in the right pelvic limb and tail 24 h after surgery. Nociception was present in the left pelvic limb after 48 h. Continued urinary incontinence necessitated manual bladder expressions in hospital and at home after discharge. The owners consulted an animal physiotherapist and started physiotherapy within 2 weeks after surgery. Slow progressive improvement in motor function was seen over the next 4 months. At reexamination during a follow-up consultation 1 month post-surgery, the cat was spastic, non-ambulatory paraparetic. Independent ambulation was not achieved over the next month, when another reexamination revealed similar findings.

Over the next 2 months, clinical deterioration and comorbidities including pyelonephritis and congestive heart failure were managed by another veterinarian. The cat was reported to have lost voluntary movement in the pelvic limbs at this point, suggesting regression to paraplegia. The patient was eventually euthanized 6 months post-surgery.

## Discussion

This case report describes the clinical findings, diagnostic imaging features including plain radiographs, plain CT, and MRI, surgical treatment, and outcome in a cat with an astrocytoma in the thoracic spinal cord. To the authors’ knowledge, there is only one previous report that describes surgical treatment and outcome for a cat with a spinal cord glioma (6). In that case, an anaplastic cervical spinal cord astrocytoma was diagnosed after surgical removal of an elliptical intraparenchymal mass at C4. The outcome in that case was positive, with complete recovery of neurological function within 3 weeks after surgery. Eventually, 4 years and 11 months post-surgery, MRI revealed recurrence of the tumor and a second surgery was performed (7). Again, a satisfactory outcome was achieved and the cat died at the age of 16 of problems unrelated to the spinal cord tumor. The outcome in the case reported here was not satisfactory, even though progress was made over the 4 months post-surgery. This likely reflects the differences in invasiveness and consistency of the mass lesions, necessitating piecemeal removal as well as increased surgical manipulation (e.g., tension on the dura mater). The latter may have put additional stress on an already injured spinal cord, negatively influencing the outcome. In the previously reported case, the surgeons were able to remove the residual mass in a large bulk, after partial piecemeal removal at first instance. Tension on the dura mater or lifting of the spinal cord was not reported in that previous case (6, 7), likely reflecting differences between the anatomically affected site as well as consistency. Another difference between our case and the case reported by Tamura et al. (6, 7), was that MRI pre-surgery subjectively revealed more perilesional edema, likely negatively influencing outcome as well.

Despite the unsatisfactory outcome as judged by the surgeon, the owners were happy with the progress that was made over the 4 months post-surgery. Further evaluation of neurological recovery or deterioration was hampered by renal and cardiac problems that ultimately led to euthanasia 6 months post-surgery. However, as neurological function recovery was minimal over 4 months post-surgery, this report provides some information on outcome of surgical treatment of spinal cord astrocytoma in this cat.

Spinal gliomas are reported infrequently in cats and typically affect adult individuals. In one previous study that reported 7 cases, a median age of 8 (range of 4–12) years was noted, and 5/7 cases were female neutered cats (5). Combining other reported feline cases (6–13), an age range between 2 and 13 years and both males and females are represented without a clear sex predisposition. In our case, a 15-year-old female neutered cat is reported. Thus, based on current data, the age range of feline patients presented for spinal cord gliomas is very broad (2–15 years). Spinal cord glioma should be considered a differential diagnosis in cats presented for (mostly chronic progressive) myelopathies. The influence of age on the prognosis regarding surgical treatment cannot be evaluated solely based on the currently available two case reports.

Reported MRI characteristics of feline spinal gliomas include intraparenchymal localization, homogenous T2W-hyperintensity, ovoid or elliptical shape, and variable contrast enhancement (none, mild, rim or ring enhancement) (5–10). An extraparenchymal appearance on MRI should not exclude glioma from the list of differentials, as this has been reported in a feline oligodendroglioma affect lumbosacral spinal cord segments (14). Notably, the authors of that case attempted surgical treatment, but intraoperative findings suggestive of intraparenchymal involvement led to a decision of euthanasia (14). Ring enhancement is regarded as a fairly classical sign of some gliomas affecting the brain of dogs or cats, but not necessarily for astrocytomas. It was reported in 1/4 cases in a case series (5), and most of the other reported cases had mild homogenous contrast enhancement. Due to the limited number of cases reported, we cannot assess if ring enhancement have any bearing on the prognosis or if it is linked to histological grade or not. Based on literature so far, it seems that an intraparenchymal localization, T2W-hyperintensity, and an ovoid or elliptical shape are the most consistent findings. In our case, all these characteristics were present. Clear ring enhancement, combined with the history of bacterial infection of the tail stump after amputation surgery, prompted a differential of spinal cord abscessation. Since CSF results were inconclusive, antibiotic therapy was initiated.

Astrocytomas are the second most common type of spinal cord neoplasia in humans, mostly affecting children (15–17). MRI is considered useful for distinguishing types of neoplasia, but distinction between the most common type of spinal cord tumor (ependymoma) and astrocytoma cannot be made reliably (15–18). Astrocytomas are reported to commonly affect multiple segments, are T1W hypo- to isointense and T2W hyperintense, and show variable contrast-enhancement with unclear margins (15–18). Ring enhancement is not a consistent feature and is has not been linked to grade or malignancy. The role of surgery in high-grade astrocytomas is controversial, though resection was favored over biopsies in some studies. Gross total removal rates are low for astrocytomas compared to other types of spinal cord neoplasia (19). Recurrence rates of spinal cord astrocytomas of up to 48% are reported (20). Interestingly, astrocytomas in the cervical spinal cord were associated with a better resectability and functional outcome (21). Again, we cannot draw conclusions based on just two case reports, but the feline case with a cervical astrocytoma and feline case with thoracic astrocytoma discussed here mirrored these findings.

Of particular note is this section in a human review article regarding surgery of spinal cord astrocytomas (17): *“Astrocytomas are more challenging than ependymomas surgically. Astrocytomas are infiltrating, have poor plane of cleavage, and blend imperceptibly with the spinal cord at the margins. Radical resection of these infiltrative lesions may result in a higher morbidity. A less radical intervention with minimal surgical morbidity is therefore preferred. In children, these tumors behave similarly to low-grade posterior fossa astrocytomas which are amenable for total resection. An “inside-out” removal is recommended based on the color and consistency of the tumor compared to the surrounding spinal cord.”*

This quote reflects some of the findings in the case reported here, including the infiltrating nature of the astrocytoma, the poor plane of cleavage, and blending with the spinal cord at the margins. The ‘inside-out’ based on color and consistency was also employed. What ‘less radical’ exactly entails is unclear, but it underlines the importance of trying to minimize further damage to healthy tissue.

One way to address the risk of damaging healthy tissue is to employ intraoperative electrodiagnostic tests, such as somatosensory evoked potentials (SEP) or motor evoked potentials (MEP) (22, 23). Indeed, the use of intraoperative monitoring techniques like this are often used and deemed critical for safe surgery of intramedullary spinal cord tumors in humans (24–28). However, to the authors’ knowledge, while these techniques have been studied in experimental feline models, there are no reports of MEP or SEP during surgery for intramedullary neoplasia in clinical small animal patients, such as dogs or cats. There are reports that document the use of such techniques for removal of extradural lesions (e.g., intervertebral disc extrusion in dogs) (29). Future studies will hopefully provide more information on the usefulness of these techniques for small animal neurosurgery.

While surgery is often the preferred treatment modality in humans, consideration can be given to radiotherapy and chemotherapy as well. Other experimental therapies in humans focus on genetic alterations in specific types of tumors and neural stem cell therapy. The reader is referred to recent reviews on these treatments for human intradural spinal cord tumors, such as Abd-El-Barr et al. (30). The use of chemotherapy or radiotherapy for intramedullary spinal cord tumors in small animals is mostly reported on a case-by-case basis.

In our case, the diagnosis was achieved based on routine histological characteristics and GFAP IHC. A validated grading system for feline astrocytoma is not available and tumors are typically subjected to the World Health Organization classification and grading system for human CNS tumors (31). The high cellularity and mitotic count in this feline astrocytoma would suggest a high-grade tumor (WHO grade 3 or 4), but definitive grading could not be determined given the small tumor area available for histologic evaluation. There are several other limitations to this case report, including the lack of a post-surgery MRI study, lack of post-mortem histopathological examination of the spinal cord, lack of further IHC tests (particularly OLIG2), and limited follow-up with regard to the spinal cord glioma-related pathology as the cat was euthanized due to concurrent disorders of other organ systems.

Nevertheless and in conclusion, this is the second case reported documenting the surgical treatment and outcome of an astrocytoma in the spinal cord of a cat. The outcome in this case was unsatisfactory with regard to neurological outcome as judged by the surgeon.

## Data availability statement

The original contributions presented in the study are included in the article/supplementary material, further inquiries can be directed to the corresponding author.

## Ethics statement

Ethical approval was not required for the studies involving animals in accordance with the local legislation and institutional requirements because the animal was treated in accordance with a high standard of care and approval by the owner. Written informed consent was obtained from the owners for the participation of their animals in this study.

## Author contributions

KS: Conceptualization, Funding acquisition, Investigation, Methodology, Visualization, Writing – original draft, Writing – review & editing. ST: Writing – review & editing. DR: Writing – review & editing. GG: Methodology, Visualization, Writing – review & editing.

## References

[1] Marioni-Henry K Vite CH Newton AL Van Winkle TJ. Prevalence of diseases of the spinal cord of cats. J Vet Intern Med. (2004) 18:851–8. doi: 10.1892/0891-6640(2004)18<851:podots>2.0.co;2, PMID: 15638269

[2] Marioni-Henry K. Feline spinal cord diseases. Vet Clin North Am Small Anim Pract. (2010) 40:1011–28. doi: 10.1016/j.cvsm.2010.05.005, PMID: 20732602 PMC7114573

[3] Mella SL Cardy TJ Volk HA De Decker S. Clinical reasoning in feline spinal disease: which combination of clinical information is useful? J Feline Med Surg. (2020) 22:521–30. doi: 10.1177/1098612X19858447, PMID: 31251096 PMC10814331

[4] Rissi DR. A review of primary central nervous system neoplasms of cats. Vet Pathol. (2023) 60:294–307. doi: 10.1177/03009858231155400, PMID: 36803009

[5] Hammond JJ deLahunta A Glass EN Kent M Summers BA Miller AD. Feline spinal cord gliomas: clinicopathologic and diagnostic features of seven cases. J Vet Diagn Investig. (2014) 26:513–20. doi: 10.1177/1040638714533118 24821692

[6] Tamura S Hori Y Tamura Y Uchida K. Long-term follow-up of surgical treatment of spinal anaplastic astrocytoma in a cat. J Feline Med Surg. (2013) 15:921–6. doi: 10.1177/1098612X13478266, PMID: 23428584 PMC11383151

[7] Tamura S Tamura Y Uchida K. Recurrence of spinal anaplastic astrocytoma in a cat after surgical treatment and long-term follow-up. J Feline Med Surg. (2018) 20:200–1. doi: 10.1177/1098612X17736658, PMID: 29065804 PMC11129267

[8] Aloisio F Levine JM Edwards JF. Immunohistochemical features of a feline spinal cord gemistocytic astrocytoma. J Vet Diagn Investig. (2008) 20:836–8. doi: 10.1177/104063870802000624, PMID: 18987242

[9] Galli G Burbaite E Rigillo A Menchetti M. Paraparesis, inappropriate urination and defecation in a 13-year-old Persian cat. J Am Vet Med Assoc. (2023) 261:1–3. doi: 10.2460/javma.22.12.0564 36735502

[10] Hasegawa D Aoshima K Sasaoka K Kobayashi A Takiguchi M Kimura T. Malignant oligoastrocytoma in the spinal cord of a cat. J Vet Med Sci. (2022) 84:1277–82. doi: 10.1292/jvms.22-0144, PMID: 35908858 PMC9523307

[11] Haynes JS Leininger JR. A glioma in the spinal cord of a cat. Vet Pathol. (1982) 19:713–5. doi: 10.1177/030098588201900618, PMID: 7147631

[12] Stigen O Ytrehus B Eggertsdottir AV. Spinal cord astrocytoma in a cat. J Small Anim Pract. (2001) 42:306–10. doi: 10.1111/j.1748-5827.2001.tb02046.x, PMID: 11440402

[13] Rissi DR Miller AD. Feline glioma: a retrospective study and review of the literature. J Feline Med Surg. (2017) 19:1307–14. doi: 10.1177/1098612X16689506, PMID: 28156189 PMC11104172

[14] Korff CP Chu SA Percival AJ Nelissen S Wood JH Davies E. Unique cytologic and imaging features of a lumbosacral oligodendroglioma in a cat. J Vet Diagn Investig. (2023) 35:289–94. doi: 10.1177/10406387231166132, PMID: 37010018 PMC10185999

[15] Do-Dai DD Brooks MK Goldkamp A Erbay S Bhadelia RA. Magnetic resonance imaging of intramedullary spinal cord lesions: a pictorial review. Curr Probl Diagn Radiol. (2010) 39:160–85. doi: 10.1067/j.cpradiol.2009.05.004, PMID: 20510754

[16] Lowe GM. Magnetic resonance imaging of intramedullary spinal cord tumors. J Neuro-Oncol. (2000) 47:195–210. doi: 10.1023/a:1006462321234 11016736

[17] Menon G Srinivasan S Nair R Hegde A Nair S. Spinal intramedullary tumors. Arch Med Health Sci. (2022) 10:247–55. doi: 10.4103/amhs.amhs_263_22

[18] Arima H Hasegawa T Togawa D Yamato Y Kobayashi S Yasuda T. Feasibility of a novel diagnostic chart of intramedullary spinal cord tumors in magnetic resonance imaging. Spinal Cord. (2014) 52:769–73. doi: 10.1038/sc.2014.127, PMID: 25091110

[19] McGirt MJ Goldstein IM Chaichana KL Tobias ME Kothbauer KF Jallo GI. Extent of surgical resection of malignant astrocytomas of the spinal cord: outcome analysis of 35 patients. Neurosurgery. (2008) 63:55–60. doi: 10.1227/01.NEU.0000335070.37943.09, PMID: 18728568

[20] Juthani RG Bilsky MH Vogelbaum MA. Current management and treatment modalities for intramedullary spinal cord tumors. Curr Treat Options in Oncol. (2015) 16:39. doi: 10.1007/s11864-015-0358-0, PMID: 26143269

[21] Ardeshiri A Chen B Hütter BO Oezkan N Wanke I Sure U. Intramedullary spinal cord astrocytomas: the influence of localization and tumor extension on resectability and functional outcome. Acta Neurochir. (2013) 155:1203–7. doi: 10.1007/s00701-013-1762-5, PMID: 23700256

[22] Costa P Bruno A Bonzanino M Massaro F Caruso L Vincenzo I. Somatosensory- and motor-evoked potential monitoring during spine and spinal cord surgery. Spinal Cord. (2007) 45:86–91. doi: 10.1038/sj.sc.3101934 16670686

[23] de Haan P Kalkman CJ. Spinal cord monitoring: somatosensory- and motor-evoked potentials. Anesthesiol Clin North Am. (2001) 19:923–45. doi: 10.1016/s0889-8537(01)80017-1 11778387

[24] Antkowiak L Putz M Sordyl R Pokora S Mandera M. Relevance of intraoperative motor evoked potentials and D-wave monitoring for the resection of intramedullary spinal cord tumors in children. Neurosurg Rev. (2022) 45:2723–31. doi: 10.1007/s10143-022-01788-2, PMID: 35416529

[25] Hersh AM Jallo GI Shimony N. Surgical approaches to intramedullary spinal cord astrocytomas in the age of genomics. Front Oncol. (2022) 12:982089. doi: 10.3389/fonc.2022.982089, PMID: 36147920 PMC9485889

[26] Hersh AM Patel J Pennington Z Porras JL Goldsborough E Antar A. Perioperative outcomes and survival after surgery for intramedullary spinal cord tumors: a single-institution series of 302 patients. J Neurosurg Spine. (2022):1–11. doi: 10.3171/2022.1.SPINE211235, PMID: 35213831

[27] Sala F Palandri G Basso E Lanteri P Deletis V Faccioli F. Motor evoked potential monitoring improves outcome after surgery for intramedullary spinal cord tumors: a historical control study. Neurosurgery. (2006) 58:1129–43; discussion 1129-43. doi: 10.1227/01.NEU.0000215948.97195.58, PMID: 16723892

[28] Roonprapunt C Houten JK. Spinal cord astrocytomas: presentation, management, and outcome. Neurosurg Clin N Am. (2006) 17:29–36. doi: 10.1016/j.nec.2005.10.006 16448905

[29] Okuno S Katahira H Orito K. Somatosensory evoked potentials of the tibial nerve during the surgical decompression of thoracolumbar intervertebral disk herniation in dogs. Front Vet Sci. (2022) 9:976972. doi: 10.3389/fvets.2022.976972, PMID: 36187812 PMC9519392

[30] Abd-El-Barr MM Huang KT Moses ZB Iorgulescu JB Chi JH. Recent advances in intradural spinal tumors. Neuro-Oncology. (2018) 20:729–42. doi: 10.1093/neuonc/nox230, PMID: 29216380 PMC5961256

[31] WHO Classification of Tumours Editorial Board. World Health Organization classification of Tumours of the central nervous system. 5th ed. Lyon: International Agency for Research on Cancer (2021).

